# Exploring Undergraduate Medical Students’ Perspectives towards Artificial Intelligence in Healthcare: A Qualitative Study from India

**DOI:** 10.31662/jmaj.2024-0402

**Published:** 2025-06-27

**Authors:** Sonali Sharma, Neha Saboo, Vaseem Naheed Baig

**Affiliations:** 1 Department of Biochemistry, RUHS College of Medical Sciences, Jaipur, India; 2 Department of Physiology, RUHS College of Medical Sciences, Jaipur, India; 3 Department of Community Medicine, RUHS College of Medical Sciences, Jaipur, India

**Keywords:** artificial Intelligence, concern, healthcare, qualitative, knowledge, machine learning, medical education, perception

## Abstract

**Introduction:**

The term “artificial intelligence (AI)” refers to the development of systems that possess intellectual processes characteristic of humans, such as the ability to reason, discern meaning, generalize, and learn from experience. This study aimed to provide an overview of medical students’ knowledge, perceptions, and concerns about AI in healthcare.

**Methods:**

This study utilized a qualitative approach, employing semi-structured interviews in focus groups of medical undergraduates to explore their perceptions, knowledge, and concerns about AI in healthcare. The interviews complied with the standards for reporting qualitative research set by the Consolidated Criteria for Reporting Qualitative Research (COREQ). Seven focus groups were formed, with an average size of 8-10 participants per group. Each group was diverse in terms of sex and year of study.

**Results:**

156 participants responded to the survey, of whom 124 completed the questionnaires. Sixty-six undergraduate medical students consented to attend in-person focus groups for discussions. Four major themes are the advantages of AI in healthcare, education, and training in AI, future implications of AI, and ethical concerns, and sixteen subthemes emerged from this study’s data analysis. Most students (57.7%) believed that artificial intelligence (AI) should be integrated into medical education. A substantial proportion (42.2%) of medical students demonstrated varying knowledge regarding the advantages of AI in healthcare. A significant number of students, 37.8%, articulated concerns regarding the future implications of AI; a minority of students, 22.7%, expressed ethical concerns regarding biases, privacy issues, security risks, and unequal access.

**Conclusions:**

Medical students generally view AI positively, recognizing its benefits in diagnosis and treatment. Many emphasized the need to integrate AI into medical education to prepare for future changes in healthcare. Future research should focus on developing evidence-based training programs and strategies to tackle these issues.

## Introduction

The term “artificial intelligence” (AI) refers to the development of systems that exhibit intellectual processes characteristic of humans, such as reasoning, discerning meaning, generalizing, and learning from experience ^(1)^. Healthcare is widely recognized as a promising field for AI applications, with significant public support for its use in this sector ^(2), (3)^. The medical field often drives technological advancements and innovations through its diverse tools, devices, and procedures.

AI, a component of the “fourth” digital industrial revolution, is assuming an increasingly significant role in contemporary healthcare ^(4)^. As AI applications are set to significantly impact medical practices, attention is now focused on the medical workforce’s readiness for this transition, particularly the perceptions of medical professionals and students ^(5)^. Challenges in implementing AI in clinical settings and bridging the clinical translation gap are well documented, partly due to clinicians’ lack of education and knowledge ^(6)^. Expertise in AI is rare among clinicians, and it is not routinely taught in medical curricula. There are calls to revise educational practices, emphasizing AI, to better prepare medical professionals for this evolving field ^(6)^.

The rapid integration of AI in healthcare necessitates an understanding of future healthcare professionals’ perspectives, particularly medical undergraduates who will lead this advancement. User perspectives are crucial for designing AI-driven educational interventions that align with the evolving medical learning landscape ^(7)^. Assessing students’ views on AI is essential to identify potential training needs, as they will regularly interact with both patients and technology ^(5)^. A systematic review by Mousavi et al. ^(8)^ on healthcare students’ attitudes toward AI revealed that while most students have a positive view of AI, they lack adequate knowledge and skills to work with these advancements.

AI is set to revolutionize the acquisition, analysis, and use of data related to patient health, healthcare services, and medical records, significantly impacting diagnostics, treatments, and research. Studies indicate that limited exposure to AI can cause anxiety among medical undergraduates and affect their career paths. Examining public perceptions and healthcare students’ knowledge can help curriculum developers identify needs in AI education ^(9)^.

A systematic review by Sapci et al. ^(9)^ found that while there are recommendations to integrate AI into the medical curriculum, this has not yet been implemented. A study reported that AI is currently absent in the medical curriculum in Kerala, India, and a majority of surveyed students expressed support for its implementation ^(10)^. A qualitative survey in India reported that medical students perceive large language models (LLMs) as potentially beneficial for medical education ^(7)^.

While research on AI in medical education is growing globally, qualitative studies on Indian medical students’ perspectives on AI are scarce. International literature has explored medical students’ attitudes toward AI in Australia ^(11)^, Palestine ^(5)^, China ^(12)^, Germany ^(13)^, Canada ^(14)^, and a study with global representation ^(15)^, providing insights into their perceptions, concerns, and readiness for AI in medical practice.

India’s rapid healthcare digitalization and diverse medical education system create a unique environment for AI adoption. Unlike documented experiences in Western and East Asian countries, Indian medical students’ perspectives on AI are largely unexplored qualitatively. Conducting qualitative studies on AI among Indian medical students fills a geographical gap in the literature, contributing to a comprehensive global understanding of medical students’ engagement with AI across diverse healthcare systems and cultural contexts. This study also provides a foundation for future comparative studies between Indian and global medical students’ perceptions of AI. This qualitative study aims to explore medical students’ knowledge, perceptions, and concerns regarding AI in healthcare.

## Materials and Methods

This study employed a qualitative approach using semi-structured interviews with focus groups of medical undergraduates to explore their perceptions, knowledge, and concerns regarding AI in healthcare.

### Ethics approval

This study was conducted in accordance with the World Medical Association’s Code of Ethics (Declaration of Helsinki) for experiments involving humans. The institutional ethical committee approved the study (reference number: RUHSCMS/Ethics Comm./2023/249, dated November 28, 2023). Participation was voluntary, and all participants provided electronic consent prior to data collection. All responses and data were kept anonymous.

### Study design

We conducted qualitative research using semi-structured interviews. The interviews followed the standards for reporting qualitative research set by the Consolidated Criteria for Reporting Qualitative Research ^(16)^.

### Study setting

This study took place at a Health Sciences University constituent medical college in India between November 2023 and March 2024.

### Sampling technique

A convenience sampling method was used to recruit participants from various study years. Eligible students were currently enrolled in the MBBS program, were 18 years or older, were willing to participate in a recorded interview, and had a basic understanding of AI in healthcare. Exclusion criteria included students from non-medical programs, postgraduate students, those unable to communicate in English or Hindi, and those who did not provide informed consent.

### Recruitment process

Potential participants were screened via a brief questionnaire to ensure they met the inclusion criteria before receiving an invitation to the interview. This process helped ensure a diverse yet relevant sample.

Initial recruitment was done through WhatsApp groups of medical students, using the snowball sampling technique to expand the pool of potential participants. We also collaborated with student representatives from various academic years to disseminate recruitment information. A recruitment link to an anonymous digital survey GOOGLE FORM was shared in WhatsApp groups for all cohorts of undergraduate medical students (approximately 850 students). The survey was presented to students after obtaining their informed consent and remained open for over 2 months, from November 2023 to December 2023.

The GOOGLE FORM was designed to collect demographic data, such as age, sex, socioeconomic status, and academic year, and to solicit participation from students familiar with AI in healthcare for focus group discussions. A total of 156 participants responded, with 124 completing the questionnaires. Sixty-six undergraduate medical students consented to attend in-person focus groups. Seven focus groups were formed, each with 8-10 participants. The groups were diverse in terms of sex and year of study.

### Designed interview protocol

The interview questions followed a semi-structured format, based on existing literature on AI in healthcare and medical education (Table 1). The questions in the study were carefully selected to ensure they addressed the research objectives, filled knowledge gaps, were relevant to participants, and had the potential to generate meaningful insights. This approach ensured that the study bridged the knowledge and perception gaps identified in the introduction.

**Table 1.** Interview Guide.

S. No.	Questions
1	Share your initial perceptions and reactions regarding the implementation of artificial intelligence in healthcare.
2	What societal implications are associated with artificial intelligence?
3	Do you have any perspectives on artificial intelligence that you would like to share?
4	In your opinion, which domains of healthcare would derive the greatest benefit from the integration of artificial intelligence?
5	In your opinion, what are the primary concerns regarding the implementation of artificial intelligence in healthcare?
6	Should artificial intelligence be incorporated into medical education curricula?
7	In your opinion, how will artificial intelligence influence healthcare over the next decade?

S. No.: serial number.

Key topics and knowledge gaps were identified through relevant studies and theoretical frameworks. The interview guide was refined through pilot testing with a small group of medical students to ensure clarity, relevance, and effectiveness in eliciting meaningful responses. Feedback from the pilot test led to revisions that improved comprehensibility and alignment with the study’s objectives. Faculty experts in medical education and qualitative research reviewed the guidelines for validity and comprehensiveness. Open-ended questions were designed to explore participants’ perceptions, experiences, and attitudes toward AI in healthcare. The protocol was developed to ensure systematic and rigorous data collection and analysis, adhering to ethical standards and enhancing the study’s credibility.

### Data collection

Semi-structured interviews were conducted to allow flexibility while maintaining consistency. Participants were informed of the interview time and date in advance. The participants were introduced to the focus group with an overview of topics and moderators. The environment was set up to foster open, candid discussions, encouraging participants to express their thoughts freely. An icebreaker activity was used to facilitate participant acclimation. Response times were equalized to ensure balanced participation.

**Figure 1.**
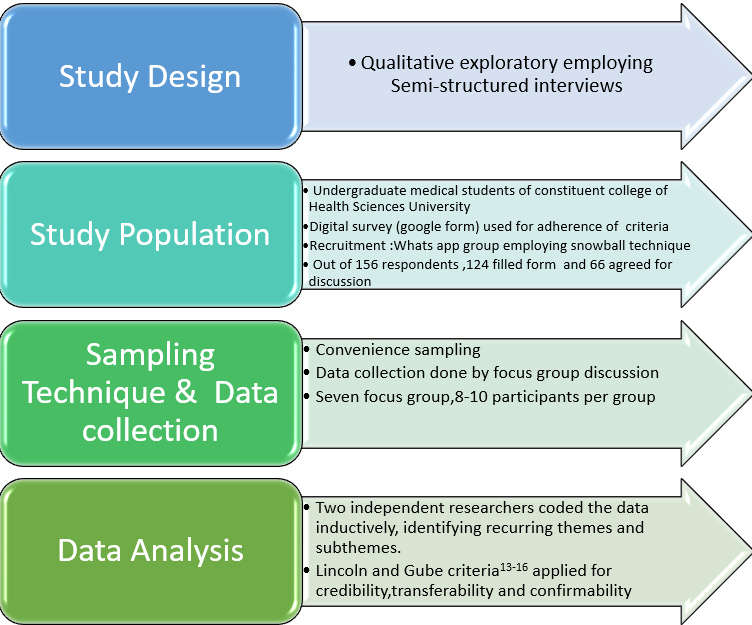
Methodology flow chart.

Before conducting the interviews, the students were reminded of the study’s objectives, methodology, and confidentiality measures. Interviews were conducted either in person or via video conferencing, with each focus group interviewed for 45-90 minutes. Following the predetermined semi-structured interview protocol, interviewees responded to relevant AI-related questions. The moderators remained neutral during discussions. The anonymity of the participants was ensured by assigning a number to each interview. The study’s methodology is depicted in Figure 1.

Two researchers collaborated on each interview; 1 conducted the session, while the other handled the recording process using a mobile device. The interviews continued until data saturation was reached, and no new themes emerged. Two investigators independently coded the data using open coding techniques until most comments could be classified under broad themes. The interviews were audio-recorded, transcribed verbatim, and analyzed using thematic analysis.

### Data analysis

Data from the GOOGLE FORM were uploaded to an Excel spreadsheet. NVivo 14 software was used for data management and coding. Thematic analysis was performed to identify, analyze, and report patterns (themes) within the data. Two independent researchers coded the data inductively and identified recurring themes. Interview transcripts were categorized and coded for content analysis. Frequency analysis was used to quantify the occurrence of specific themes or concepts across interviews. Member checking ensured trustworthiness, and an audit trail was maintained throughout the analysis. Lincoln and Guba criteria ^(17), (18), (19), (20)^ were applied to ensure credibility, transferability, dependability, and confirmability, ensuring the study’s rigor. The dataset was complete, with no missing information.

## Results

**Figure 2.**
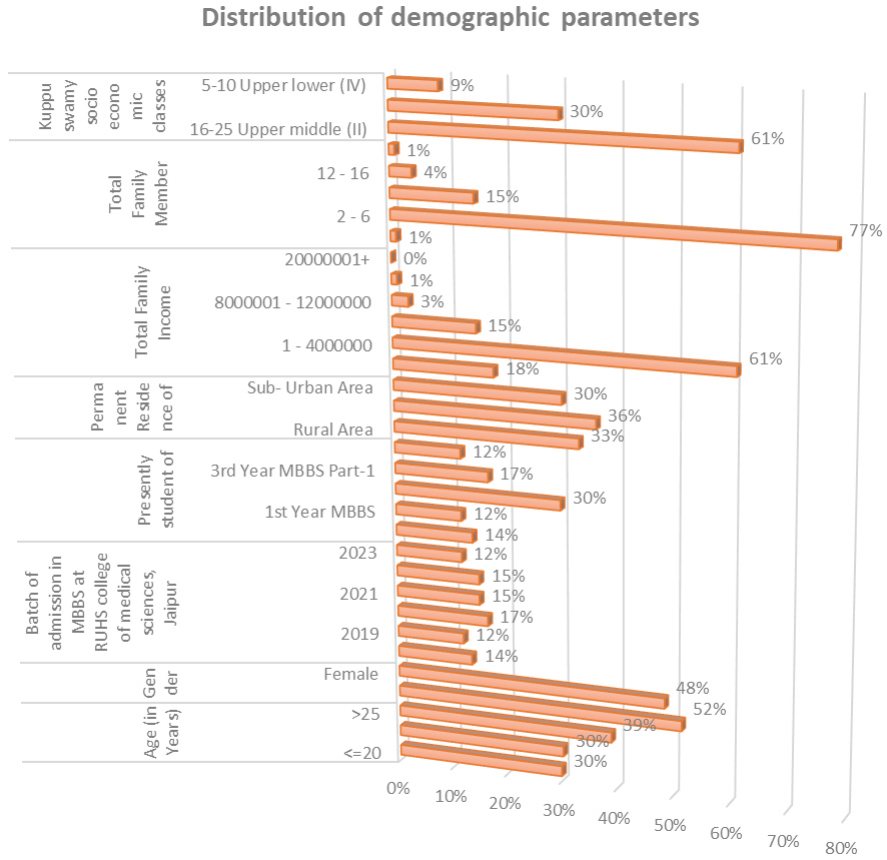
Sociodemographic variables of study participant.

The study included 66 participants, with a response rate of 53.22%. The sex distribution was relatively balanced. The sociodemographic variables are summarized in Figure 2. The majority of participants (57.3%, n = 3) were 20 years old. The sex distribution was nearly even, with 52% (n = 34) male and 48% (n = 32) female. The largest group consisted of second-year MBBS students (30%, n = 20), predominantly from urban areas (36%, n = 20). A significant proportion (61%, n = 40) had a family income ranging from 1 to 4 million, and 77% (n = 51) had 2-6 family members. The predominant socioeconomic group was the upper middle class (II) (61%, n = 40).

Figure 2 shows the sociodemographic variables of study participants and the data analysis revealed 6 main themes and 18 subthemes.

### Theme 1: Advantages of AI in healthcare (Table 2)

#### Sub-themes

*AI as a tool in medicine*: Medical students in this study viewed AI as a valuable tool, acknowledging its role in enhancing diagnostic accuracy, personalizing treatment plans, and improving efficiency in various medical processes, including treatment of rare diseases, and genetic disorders.

**Table 2.** Perspectives on Artificial Intelligence in Healthcare: Thematic Analysis and Representative Comments.

Theme	Count Percent	Sub-Themes	Representative Comments
Advantages of AI in Health Care	28 (42.2%)	AI as a tool in medicine	*“AI is helpful in diagnosing and generating/prescribing Non-communicable disease (in personalized treatment plans)”.*
			*“AI can be helpful in readings MRIs CTs X rays etc. and detecting lung cancer or strokes based on CT scans”*
			*“AI shows promise in aiding diagnosis, treatment, and management across various diseases.”*
			*“AI can assist in diagnosing rare diseases by analyzing symptoms, genetic data, and medical literature”*
		Computer Science	*“AI algorithms can analyze large datasets of patient symptoms”.*
		Positive perceptions	*“AI can always help and collaborate with human intelligence as AI itself is an invention of human intelligence.”*
			*“Yes, it is inevitable, the future; how to best use AI to enhance patient care will give an edge.”*
			*“There will be more chances for the availability of health services to all.”*
			*“It might reduce chances of human errors, easy handling of data and fast access to data, enhance and improve medical research by compiling more and more observations, etc.”*
Education and Training in AI	38 (57.7%)	Adapting to Advancements	*“Yes, because it will help us level up and be prepared for the future in which incorporation of AI is inevitable.”*
			*“Yes, because the upcoming world is of AI and we have to adopt accordingly."*
			*“Integration and Training: The ease of integrating AI technologies into existing healthcare systems and the availability of adequate training for healthcare professionals and students are crucial.”*
		Medical curricula should include AI	*“I think as a student, AI would be beneficial for better medical education with 3D models and animations.”*
			*“Yes it should be included but it should be practical based not just a theory lecture.”*
			*“Yes, to understand the basic concept and also can perform research for more better uses of AI in the medical field.”*
			*“Yes, I believe because many healthcare sectors had incorporated AI technologies already, so for better understanding and clinical expertise of students with AI technology it must be a part of curriculum.”*
		Transparency & Explainability	*“Ensure transparency in AI algorithms and hold parties accountable.”*
Future Implications of AI	25 (37.8%)	Easy workflow	*“AI has the potential to streamline administrative tasks, optimize workflows, and reduce manual labor.”*
			*“AI shows promise in aiding diagnosis, treatment, and management across various diseases. Some specific areas where AI has demonstrated potential include: Alzheimer’s disease, Parkinson’s disease, and stroke and lung cancer*
			*“Detection of new pandemic or epidemic threats,”*
		Potential cost saving	*“Statistics and data analysis will be easy by AI.”*
			*“AI has the potential to bring cost savings through improved efficiency in healthcare delivery.”*
			*“AI can reduce patient cost merely due to the virtue of faster and more efficient mechanism of functioning in the long term”*
		Relevance to healthcare	*“AI in healthcare is like exploring the new world for the healthcare system, education, and practices.”*

AI: artificial intelligence; CT: computed tomography.

*Computer science:* Participants highlighted AI’s potential to manage complex data analysis tasks and support medical decision-making across a wide range of conditions and specialties.

*Positive perceptions:* Quotes from medical students in this qualitative study emphasized the synergistic potential of combining AI’s computational power with human expertise and judgment. They viewed AI as a contemporary tool, recognizing its inevitability in healthcare. Students acknowledged that AI’s ability to compile and analyze vast amounts of observational data could drive medical research forward. These perspectives reflect an optimistic outlook, highlighting AI’s potential to enhance human capabilities, improve patient care, and foster innovation in healthcare.

### Theme 2: Education and training in AI (Table 2)

#### Sub-themes

*Adapting to advancements:* Participants recognized the inevitability of AI integration across various fields, including healthcare. They emphasized the importance of education and training to “level up” and prepare for an AI-driven future. They stressed the need for comprehensive education and training programs for current healthcare professionals, ensuring they are prepared for an AI-integrated future and can develop solutions that integrate effectively into existing systems.

*Medical curricula should include AI:* Participants discussed strategies to incorporate AI into the medical curriculum. The highlighted AI’s potential to enhance medical education through advanced visualization techniques like 3-dimensional models and animations, providing engaging and effective learning tools. They emphasized the need for practical, hands-on experience with AI and the importance of designing curricula that incorporate real-world applications in healthcare. Students recommended integrating AI technologies across healthcare sectors to keep medical education current, while ensuring a balanced approach combining theoretical knowledge, practical application, and research opportunities.

*Transparency and explainability:* Medical students stressed the necessity for explainable AI, ensuring transparency in AI systems and enabling users and stakeholders to understand the decision-making processes.

### Theme 3: Future implications of AI (Table 2)

#### Sub-themes

*Easy workflow:* Students expressed that AI has the potential to optimize workflows and reduce manual labor, leading to more efficient operations across various sectors. They marveled at AI’s capabilities in enhancing medical diagnosis, treatment, and management across multiple diseases, potentially improving accuracy and speed. Students also highlighted AI’s role in detecting pandemics and epidemic threats, improving global preparedness for health crises.

*Potential cost saving:* Participants discussed the potential of AI to automate and simplify complex analytical tasks, which could lead to significant cost savings, such as streamlined patient care processes and more efficient and accurate diagnoses.

*Relevance to healthcare:* Participants highlighted key future implications of AI in healthcare. One participant likened AI’s role in healthcare to “exploring the new world,” suggesting that AI could fundamentally reshape healthcare systems, education, and practices. Students discussed AI’s capacity to streamline complex tasks and addressed its economic implications, including the potential for reduced healthcare costs at both institutional and patient levels.

### Theme 4: Concerns related to the usage of AI in healthcare in a negative perspective (Table 3)

#### Sub-themes

*Data security and social threat:* In this study, participants highlighted concerns about AI systems potentially being vulnerable to hacking or manipulation, which could compromise sensitive data or critical infrastructure. Participants emphasized AI’s potential to be weaponized for harmful activities.

**Table 3.** Challenges and Concerns of Artificial Intelligence in Healthcare: Thematic Analysis and Representative Comments.

Theme	Count Percent	Subthemes	Representative Comments
Concerns related to use of AI in healthcare	15 (22.7%)	Data Security and Social Threat	*“AI can be a social threat due to potential biases, privacy concerns, security risks, and unequal access."*
			*“AI can be a social threat, because people are misusing the ability of AI to defame others, cheating, and frauds, etc."*
			*“Yes, it is a social threat because AI doesn't preserve someone's private data and makes it public to everyone."*
			*“Yes, it can be easily misused to retract information about patients and result in the breach of their privacy."*
		Emotional Intelligence	*“While AI systems can perform specific tasks with remarkable efficiency and speed, they lack the broad cognitive abilities, emotional understanding, and creativity that characterize human intelligence."*
			*“AI can replace humans in terms of mental intelligence but not in the case of emotional intelligence; both are required to deal with patients."*
		Economic Consideration	*“Initial Costs: Implementing AI technologies... can initially increase the expenditure for hospitals."*
			*“AI may initially increase hospital setup costs... For hospital set up, the expenditure will increase potentially leading to higher service fees."*
		Ethical concerns	*“Educating both healthcare professionals and the public about the ethical implications of AI in healthcare is lacking.*
			*“To address ethical and privacy issues, clear regulations are lacking, ensure transparency in decision-making, prioritize secure and anonymized data handling, and promote ongoing public education."*
			*“AI raises ethical questions regarding decision-making, accountability, and transparency."*
			*“AI is a threat, they are machines, they are much more capable than us at various tasks, and maybe at strength too; they can cause job replacements, depression in humans, leading to the total failure of humanity."*
Job displacement	35.44%	Healthcare automation	*“Machines taking over in surgeries and certain AI apps providing diagnosis are my initial thoughts. This can be a threat to jobs for humans in healthcare.”*
			*“It can be possible in the future as AI is becoming smarter by each passing days’*
			*“While AI is expected to augment human capacities, there is fear that increasing dependence on machine-driven networks will reduce our capacity for independent thinking, our social skills, and our ability to take decisions without an automated system."*
			*“Yes, AI is going to be the reason for a loss of a lot of jobs”*
		Economic impact	*“Doctor can be replaced in the future because overdependence on AI is growing.”*
			*“The automation capabilities of AI have the potential to replace certain jobs, leading to concerns about unemployment and economic inequality.”*
			*“Yes, it will surely replace them there will be no human intellectual between each other”*
			*“AI is going to be the reason for the loss of a lot of jobs.”*
Patient Interaction and Human Touch	42.66%	Risk of self-diagnosis and AI-generated medical advice	*“Doctor Google, people are risking their health by complying with AI-generated treatment available on Google”*
		Impact on patient-doctor relationship	*“In healthcare, there is always going to be a need for human touch, thinking, and spontaneity”*
			*“No in medical sciences, patient care requires a humanized touch”*
			*“It might cause. weakening of doctor-patient relations.”*
		Surgical errors by AI	*“AI is just software; it has advantages and disadvantages, but just an error during surgery can risk a life. So, I don't agree about robotic surgery.”*

AI: artificial intelligence.

*Emotional intelligence*: In this qualitative study, participants foresaw a future where AI and human intelligence complement each other, rather than AI fully replacing human roles. They attributed this to AI’s lack of broader cognitive abilities and the absence of a human touch. Students stated that AI lacks emotional intelligence, which highlights a significant limitation, especially in fields like healthcare, where empathy and emotional understanding are crucial.

*Economic considerations*: Medical students suggested that implementing AI technologies would increase initial expenditures for healthcare facilities. This indicates that healthcare institutions need to prepare for significant upfront investments when adopting AI systems. Students emphasized the need to balance the potential benefits of AI with the financial challenges of its adoption.

*Ethical concerns:* Participants highlighted a critical gap in knowledge regarding the ethical aspects of AI implementation in healthcare. Students indicated insufficient training for healthcare professionals on AI ethics and limited public understanding of AI’s implications. Participants also emphasized the lack of clear regulations to address ethical and privacy issues in AI healthcare applications, which may lead to inconsistent practices and potential misuse of AI. In this study, students perceived AI as a potential threat to human society, likely due to its rapid advancement and unpredictable future developments. Participants acknowledged that AI surpasses human abilities in various tasks, which could lead to feelings of inadequacy or obsolescence among humans. The concerns extended to psychological impact and existential threats, reflecting fears about the loss of human control and potential extinction.

### Theme 5: Job displacement (Table 3)

*Healthcare automation*: This research discovered that AI and machines are perceived as potential threats to human healthcare jobs, particularly in surgery and diagnosis. Participants expressed that AI is progressing rapidly and viewed the scenario of machines taking over healthcare roles as plausible, though not an immediate reality. In this study, participants voiced concerns about over-reliance on machine-driven systems, which could reduce human skills. The statements suggest a need to consider the long-term effects of AI integration on healthcare quality, accessibility, and the future of the medical profession.

*Economic Impact*: Participants believe that AI could replace many jobs, including specialized roles like doctors, and view growing AI dependence as a threat to employment. Some participants expressed concerns about widespread unemployment and economic inequality due to AI adoption. While some see job displacement as a future possibility, others view it as inevitable. Students implicitly worry that human skills and expertise may become less valuable as AI advances. These perspectives highlight the need for careful consideration of AI integration, balancing technological advancement with employment stability and human expertise.

### Theme 6: Patient interaction and human touch (Table 3)

#### Sub-themes

*Risk of self-diagnosis and AI-generated medical advice*: In this study, participants raised concerns about the “Doctor Google” phenomenon, where individuals attempt to diagnose themselves using online resources. They emphasized the dangers of relying on AI-generated treatments found on the internet and the potential health risks associated with it. This sub-theme underscores the importance of professional medical guidance over AI-generated advice.

*Impact on patient-doctor relationship*: Medical students reflected on the importance of human touch in healthcare settings and expressed concerns about AI potentially replacing human interaction in medical contexts. They feared that increased reliance on AI could weaken the interpersonal aspects of healthcare delivery.

*Surgical errors by AI*: In this research, some participants expressed concern about the potential for surgical errors by AI systems, which could have life-threatening consequences. Additionally, some participants expressed skepticism toward robotic surgery, indicating a preference for human-performed procedures.

## Discussion

This study employed a qualitative approach, utilizing interviews in focus groups of medical undergraduates to explore their perceptions, knowledge, and concerns regarding AI in healthcare.

Six major themes and 18 subthemes emerged from the data analysis. The qualitative analysis indicates that medical students show varying levels of knowledge about AI’s advantages in healthcare. Their perception of AI as a tool for diagnostics, analysis, and treatment prediction aligns with Stewart et al. ^(11)^, who found that students believe AI should be integrated into medical education and healthcare systems.

Clinicians must understand AI and its role in healthcare. The findings of this study align with those of Fazakarley et al. ^(21)^, who reported in a qualitative survey among healthcare staff that AI tools were also anticipated to enhance diagnostic accuracy, minimize human error, and reduce clinician workload. The findings of this investigation also align with earlier work by Morrison ^(22)^, who conducted a qualitative analysis of expert opinions. Their research revealed that AI tools were viewed as capable of assisting clinical staff and enhancing their work environment.

Funer et al. ^(23)^ conducted a qualitative interview study to explore medical students’ perceptions of ethical issues surrounding AI-driven Clinical Decision Support Systems (AI-CDSS), with a particular emphasis on the knowledge and competencies required to utilize such systems in clinical practice. The study’s findings contribute to the discourse on essential competencies, appropriate training programs, and professional standards necessary for the safe and effective clinical implementation of AI-CDSS across various medical fields.

Medical students generally exhibit positive perceptions of AI in healthcare, recognizing its potential benefits while also expressing concerns. A scoping review of health science students’ perceptions, including medical students, found overall positive attitudes toward AI's potential benefits in their future work. Students showed interest in and willingness to learn about AI, although concerns were raised about job security and the lack of realism in AI software ^(24)^.

In this study, many students believed AI should be integrated into medical education due to its growing role in healthcare, emphasizing the need for adequate preparation. This aligns with Jebreen et al. ^(5)^, who found that incorporating AI training into medical curricula is crucial, given AI’s rapid advancement and increasing use in medicine worldwide. Wartman and Combs ^(25)^ concluded that students should be taught medical practice in a context where AI has revolutionized the field.

Mir et al. ^(26)^ argued in their review that curriculum development, learning, and assessment are key areas where AI can be utilized in medical education. AI has the potential to reduce the time required for reviewing curricula, address complex problems, enhance classification accuracy, and elucidate relationships between parameters in curriculum assessment.

The results align with Mondal et al. ^(7)^, who found that students perceive LLMs as beneficial for medical education. However, optimizing LLM integration requires addressing their challenges and strengths. Shaima et al. ^(27)^ conducted a qualitative exploratory study, where most participants suggested revising the medical curriculum to include AI-based medical education and fundamental computer education.

The findings of this study align with another review by Dave and Patel ^(28)^, which posits that AI can complement and augment human tasks by facilitating remote learning for students and faculty in areas with limited human resources, thereby impacting both academia and healthcare.

Health science students are critical in developing and applying AI to healthcare. The theoretical knowledge and practical applications of AI span various health disciplines, and students’ perceptions and attitudes regarding AI are diverse. From a practical standpoint, educators should prioritize providing evidence‐based examples of AI, incorporating hands‐on training, industry input, promoting continuous professional development, and fostering interprofessional collaboration ^(24)^.

Many participants in this study voiced concerns about AI’s future implications. They highlighted AI’s role in improving workflow efficiency by minimizing manual labor. AI has reduced costs and enhanced efficiency in healthcare delivery. Participants likened AI in healthcare to exploring new frontiers for healthcare systems, education, and practices. These findings align with previous studies ^(21), (29)^.

Medical students generally demonstrate positive perceptions of AI in healthcare, recognizing its potential benefits while also expressing some reservations. Several qualitative studies have explored this topic in depth. A scoping review of health science students’ perceptions, including medical students, revealed favorable attitudes toward AI’s potential advantages in their future careers. Students exhibited interest and a willingness to learn about AI, although concerns were raised regarding job security and the perceived lack of realism in AI software ^(30)^.

In the present study, a smaller proportion of students expressed ethical concerns regarding biases, privacy issues, security risks, and unequal access. They emphasized the need to educate healthcare professionals and the general public about the ethical implications of AI in healthcare. Participants in this study raised concerns about AI tools related to data protection and patient privacy, aligning with the survey conducted by Fazakarley et al. ^(21)^, who reported that data security is not unique to AI tools.

Students in medical programs expressed concerns about ethical issues related to academic honesty, data protection, copying without attribution, and the lack of cultural awareness in AI systems ^(31)^. Weidener et al. ^(32)^ determined that ethical considerations, privacy issues, and regulatory frameworks should be incorporated into the use of AI in medical training. The research by Chan and Zary ^(33)^ also highlighted the ethical dilemmas of integrating AI into medical education, particularly in teaching empathy.

These results are consistent with studies investigating the acceptability of various technological advances in healthcare over the years, including emails, mobile healthcare applications, and remote healthcare consultations ^(31), (32), (33)^.

In a quantitative survey, Jha et al. ^(34)^ reported that the systematic implementation of AI in the healthcare system could address challenges related to resource and manpower constraints. Incorporating AI and machine learning into medical curricula may serve as an advantageous initial approach. These findings are consistent with those of the present study. AI-driven technologies are poised to transform medical education by helping educators understand student needs, address individual learning patterns, and enable institutions to emphasize communication, ethics, and morals.

Although bias in AI-driven educational frameworks is a concern, ongoing research is expected to significantly mitigate this issue, fostering a more equitable learning environment. Educators should prioritize evidence-based AI examples, hands-on training, industry input, continuous professional development, and interprofessional collaboration.

Educational institutions should implement structured collaborative learning experiences that integrate health and data science training, while introducing an ethical framework for AI consideration. Concepts should focus on enduring core principles of AI without delving into technical details. Institutions should create opportunities for medical students to engage with AI professionals from the health industry. Learner engagement should be facilitated through practical workshops that emphasize the development of AI-related skills.

The strength of our study lies in its qualitative method, which provides an in-depth and nuanced understanding of complex phenomena related to AI in healthcare among undergraduate medical students. This approach captured subtleties and contextual factors, offering comprehensive narratives and real-world perspectives. By examining key areas of AI in healthcare within a broader context, the study’s open-ended nature allowed for unexpected findings. Our study has some limitations. The distinct characteristics observed across academic years were not explored, and we recommend incorporating this analysis in future research or as an extension of the current study. This analysis could be essential for designing more targeted and effective AI-related curriculum interventions and determining the optimal timing for introducing AI-related education in the medical curriculum. Subjective data interpretation may introduce researcher bias, potentially affecting the findings. The limited sample size restricts generalizability of the results to a larger population; a more extensive multicentric interview approach would have been beneficial. Additionally, qualitative research lacks the statistical rigor of quantitative methods, making it challenging to establish cause-and-effect relationships or definitively test hypotheses. Nevertheless, our detailed analysis yielded reliable outcomes and maintained validity.

### Conclusion

The findings indicated that medical students generally exhibit positive attitudes toward AI, recognizing its potential benefits in diagnosis, analysis, and treatment. Many participants emphasized the importance of integrating AI into medical education to prepare future healthcare professionals for the evolving landscape of medicine. While students acknowledged AI’s potential to enhance efficiency, reduce errors, and improve healthcare delivery, they also expressed concerns about data privacy, security risks, and potential job displacement.

These findings highlight the need for comprehensive AI education in medical curricula. As AI revolutionizes healthcare, medical education must adapt to equip future professionals with the skills and knowledge needed to effectively use AI technologies while upholding ethical standards and patient-centered care.

Recommendations for medical educators include integrating AI-focused modules into the core medical curriculum, developing hands-on training programs for practical AI applications in healthcare, organizing regular workshops and seminars on emerging AI technologies, and collaborating with AI experts to create interdisciplinary learning opportunities. Furthermore, recommendations for policymakers include allocating funds for AI education and training programs in medical schools, establishing guidelines for the ethical AI use in healthcare settings, creating incentives for healthcare institutions to adopt AI technologies responsibly, and developing a national framework for AI literacy in medical education. There is also a need to address implementation challenges. Future research on the long-term impact of AI education on clinical outcomes and the effectiveness of various teaching methodologies in medical education is warranted. Suggested potential pilot interventions for incorporating AI education into medical curricula include introducing AI modules, AI-enhanced case studies, hands-on workshops, ethics seminars, research projects, guest lectures by AI experts (inviting industry professionals or researchers to share insights on AI advancements in medicine), and AI-focused electives.

By conducting qualitative studies on AI among Indian medical students, this research not only fills a geographical gap in the literature but also contributes to a more comprehensive global understanding of medical students’ engagement with AI across diverse healthcare systems and cultural contexts. Further research in diverse medical education settings is recommended to validate and expand these findings.

## Article Information

### Conflicts of Interest

None

### Acknowledgement

We express our sincere gratitude and thanks to the Medical Education Unit of Pramukhswami Medical College (PSMC), Karamsard, Gujarat (India).

### Author Contributions

Conceptualization, Study Design, and Manuscript Editing: Sonali Sharma; Definition of intellectual content: Vaseem Naheed Baig, Neha Saboo; Literature Search, Data Analysis, and Manuscript Preparation: Sonali Sharma, Neha Saboo; Data Acquisition: Sonali Sharma, Vaseem Naheed Baig. All authors read and approved the final manuscript.

### Informed Consent

Informed consent was obtained from all participants who agreed to participate in the study.

### CTRI Registration Number

CTRI no: CTRI/2024/05/066751
